# An assessment of intra-patient variability on observed relationships between wall shear stress and plaque progression in coronary arteries

**DOI:** 10.1186/1475-925X-14-S1-S2

**Published:** 2015-01-09

**Authors:** David S Molony, Lucas H Timmins, Olivia Y Hung, Emad Rasoul-Arzrumly, Habib Samady, Don P Giddens

**Affiliations:** 1 Wallace H. Coulter Department of Biomedical Engineering, Georgia Institute of Technology and Emory University, Atlanta, GA, 30332, USA; 2 Division of Cardiology, Department of Medicine, Emory University School of Medicine, Atlanta, GA, 30322, USA

## Abstract

**Background:**

Wall shear stress (WSS) has been associated with sites of plaque localization and with changes in plaque composition in human coronary arteries. Different values have been suggested for categorizing WSS as low, physiologic or high; however, uncertainties in flow rates, both across subjects and within a given individual, can affect the classification of WSS and thus influence the observed relationships between local hemodynamics and plaque changes over time. This study examines the effects of uncertainties in flow rate boundary conditions upon WSS values and investigates the influence of this variability on the observed associations of WSS with changes in VH-IVUS derived plaque components.

**Methods:**

Three patients with coronary artery disease underwent baseline and 12 month follow-up angiography and virtual histology-intravascular ultrasound (VH-IVUS) measurements. Coronary artery models were reconstructed from the data and models with and without side-branches were created. Patient-specific Doppler ultrasound (DUS) data were employed as inflow boundary conditions and computational fluid dynamics was used to calculate the WSS in each model. Further, the influence of representative coronary artery flow waveforms upon WSS values was investigated and the concept of treating WSS using relative, rather than actual, values was explored.

**Results:**

Models that included side-branch outflows and subject-specific DUS velocities were considered to be the reference cases. Hemodynamic differences were caused by the exclusion of side-branches and by imposing alternative velocity waveforms. One patient with fewer side-branches and a scaled generic waveform had little deviation from the reference case, while another patient with several side-branches excluded showed much larger departures from the reference situation. Differences between models and the respective reference cases were reduced when data were analyzed using relative, rather than actual, WSS.

**Conclusions:**

When considering individual subjects, large variations in patient-specific flow rates and exclusion of multiple side-branches in computational models can cause significant differences in observed associations between plaque evolution and ranges of computed WSS. These differences may contribute to the large variability typically found among subjects in pooled populations. Relative WSS may be more useful than actual WSS as a correlative variable when there is a large degree of uncertainty in flow rate data.

## Background

Wall shear stress (WSS) derived from computational fluid dynamics (CFD) has emerged as a potential tool to predict coronary artery plaque progression in humans. The PREDICTION study demonstrated that segments exposed to low WSS resulted in an increase in plaque burden [1] and a study by Samady *et al*. also found an increase in plaque burden in low WSS regions [2]. Further, a decrease in plaque area accompanied with an increase in necrotic core area was observed in regions exposed to high WSS - suggestive of a transformation to a vulnerable plaque phenotype [2]. As well as these prospective studies, there have been several cross-sectional studies that have shown similar results. Early plaques have been found to have a greater degree of necrotic core exposed to low WSS while advanced plaques (plaque burden > 40%) have a higher degree of necrotic core exposed to high WSS [3]. After adjusting for plaque burden Eshtehardi *et al*. also found necrotic core to be greater in low WSS segments [4].

In order to predict WSS precisely in human coronary arteries accurate measurements of patient geometry and flow are necessary. Both of these measurements can be made through interventional techniques; patient geometry by a combination of intravascular ultrasound (IVUS) and angiography, and flow through the use of Doppler ultrasound (DUS). It has previously been shown that coronary artery geometry is the most important determinant of WSS patterns [5]; and although cardiac motion alters coronary artery geometry, this has been found to have little effect on time averaged WSS calculations [6,7]. However, the presence of side-branches has not been fully addressed. Local WSS patterns have been shown to be altered by the presence of side-branches, and differences of up to 12 Pa have been reported in arteries with multiple side-branches [8,9]. The impact of these hemodynamic differences on observed relationships of WSS and plaque progression has not been investigated to date. Further, it has been shown that higher inlet flow rates elevate WSS magnitudes [10]. As patients are at rest and typically sedated during catheterization, velocity measurements represent only a snapshot of the patient's coronary flow, raising the question of relying upon this one-time measurement when coronary flow rates can be expected to vary throughout a normal day. Given the large variability than can be seen in an individual patient, these factors may have an important impact on actual WSS values.

This study investigates the sensitivity of observed relationships between computed values of WSS and changes in plaque composition over time by altering two parameters. Firstly, we look at the impact of excluding coronary side-branches on WSS calculations, an assumption made in some studies [1,3]; and secondly, we investigate the effect of using physiologically representative velocity waveform inlet data rather than patient-specific velocities. Because of expected variability in velocity/flow boundary conditions, we also introduce the concept of relative WSS in examining WSS/plaque changes relationships.

## Methods

### Data acquisition

Three patients were randomly selected from a group who presented to the Cardiac Catheterization Laboratory at Emory University Hospital with stable angina or an abnormal non-invasive stress test and were found to have a non-obstructive lesion. At baseline, all patients underwent biplane coronary angiography and VH-IVUS (20 MHz Eagle Eye® Gold Catheter, Volcano Corp., Rancho Cordova, CA) image acquisition. Pressure and velocity measurements were recorded using a 0.14 inch pressure and Doppler flow velocity monitoring guidewire (ComboWire®, Volcano Corp., Rancho Cordova, CA). Patients returned to the catheterization laboratory approximately 12 months after initial catheterization and repeat imaging was performed. All human data used in this study were obtained from consenting patients who presented to the Cardiac Catheterization Laboratory at Emory University Hospital and was approved by the hospital's Ethical Research Committee. Patient data related to the arteries investigated are given in Table 1.

**Table 1:** Patient geometry characteristics.

	1	2	3
Vessel	Left main + LAD	LAD	LAD
No. side branches	3	2	5
Vessel length (mm)	57	34	57
Mean inlet velocity (m/s)	0.18	0.26	0.2
Baseline plaque burden (%)	49	37.3	49.7
Follow-up plaque burden (%)	41.8	42.6	58.1
Baseline lumen area (mm^2^)	6.28	6.62	7.3
Follow-up lumen area (mm^2^)	7.95	5.67	6.83

Mean inlet velocity is the time averaged inlet velocity. Plaque burden is defined as vessel cross-sectional area (CSA) minus luminal CSA divided by luminal CSA. Values for lumen area and plaque burden area measured at each VH-IVUS image and averaged over the entire vessel.

### Computational modelling

Three-dimensional reconstruction of coronary arteries was achieved by combining biplane angiography data with VH-IVUS images. This process has previously been described extensively [11-13]. Briefly, the 3D spatial location of the IVUS catheter prior to pullback is determined via back-projection (IC-PRO, Paeion Inc., Ha'ayin, Israel), and this serves as a backbone for stacking the IVUS images, which are placed perpendicular to the catheter with the distance between consecutive images determined by the pullback speed (0.5 mm/s). Coronary side-branches are then identified in the images and added to the main vessel reconstruction. Initially, we attempted to extract this information from biplane angiography data, but it was often not possible to view a side-branch simultaneously in both angiographic images. In cases where side-branches were visible in both views and 3D reconstruction was possible, the take-off point was frequently deemed to be anatomically incorrect. For these reasons side-branches were modelled as cylindrical extensions normal to the centreline of the main vessel, and the side-branch take-off location and orientation were obtained from the IVUS images.

**Figure 1:**
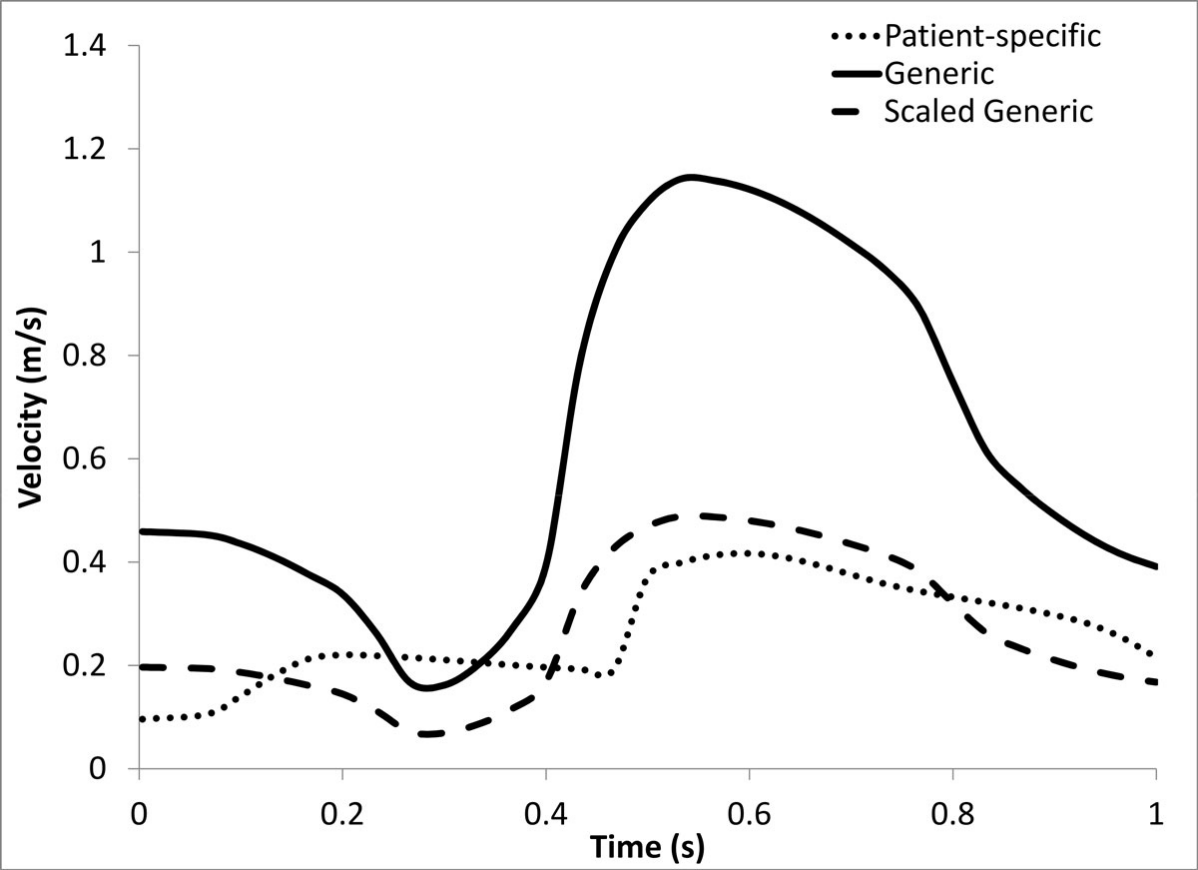
**Patient-specific, generic, and scaled generic velocity waveforms for patient 2**. The generic waveform is taken from another patient in our database and gives a mean flow rate for the LAD of 208 ml/min, while the scaled generic waveform has the same shape but results in a mean flow rate of 89 ml/min, which is the value measured for patient 2.

Models were meshed with hexahedral elements (ICEM-CFD, ANSYS Inc., Canonsburg, PA) and flow extensions were added to the inlet and outlet boundaries. CFD simulations were performed using Fluent (ANSYS Inc., Canonsburg, PA) with 300 time steps per cardiac cycle. Patient velocity data acquired *in vivo *were used to compute flow waveforms that were imposed as inlet boundary conditions, while the outlet boundaries were assigned zero outlet pressure. Blood was assumed to be an incompressible Newtonian fluid with a density of 1050 kg/m^3 ^and viscosity of 0.0035 Pa s. Simulations were performed for each patient including and excluding side-branches and using the patient-specific DUS velocity measurements for inlet flow conditions. To explore effects of variations in inflow, further simulations were performed on patient 2 using a physiologically representative inflow velocity waveform (Figure 1) obtained from another patient in our database (termed "generic waveform") and also using this waveform scaled to match the measured mean flow rate of patient 2 (termed "scaled generic waveform").

### Data analysis

Given that atherosclerosis is a focal disease we investigated WSS and plaque composition changes in 45 degree sectors of each IVUS image [13]. Firstly, we co-registered baseline VH-IVUS images with follow-up VH-IVUS images. Using fiduciary anatomical landmarks such as side-branches, images were co-registered in the axial direction. Next, images were circumferentially co-registered through normalized cross-correlation by taking into account plaque thickness, plaque composition and perivascular tissue [14]. After circumferential co-registration was complete, plaque composition changes from baseline to follow-up were quantified in 45 degree sectors. VH-IVUS identifies four different components of plaque, namely Fibrotic (FB), Fibro-fatty (FF), Necrotic core (NC) and Dense Calcium (DC).

Baseline time-averaged wall shear stress data were then associated with baseline plaque components in the 45 degree sectors, resulting in a baseline WSS value, baseline plaque component area, follow-up plaque component area, and change in plaque component area for each sector. Mean plaque component area changes were then placed into low (WSS < 1 Pa), intermediate (WSS 1-2.5 Pa) and high (WSS > 2.5 Pa) WSS categories depending on the WSS value (2). Due to the potential variation in patient data we repeated the analysis using relative WSS, which we defined by dividing all WSS values into 20 equally sized bins. These values and their associated plaque component area changes were then classified into low, intermediate and high WSS categories. Mean plaque component area change in each category was then reported.

Statistical analysis was performed using Microsoft Excel. Agreement in WSS between models with and without side-branches or using alternative waveforms was assessed by Bland-Altman analysis. This method is used to assess whether there is a consistent bias in WSS calculation from a given method. Continuous variables are reported as mean ± 95% confidence interval. An unpaired Student's *t*-test was used to investigate the association of continuous (e.g., change in plaque area) to categorical (e.g., WSS category) variables. Comparisons were made between changes in VH-IVUS defined constituents in WSS categories within the two analysis methods (e.g., side-branches included or excluded) for each patient. All statistical tests were two-tailed, and *P *< 0.05 was established as the level of statistical significance.

## Results

### Hemodynamics

**Figure 2:**
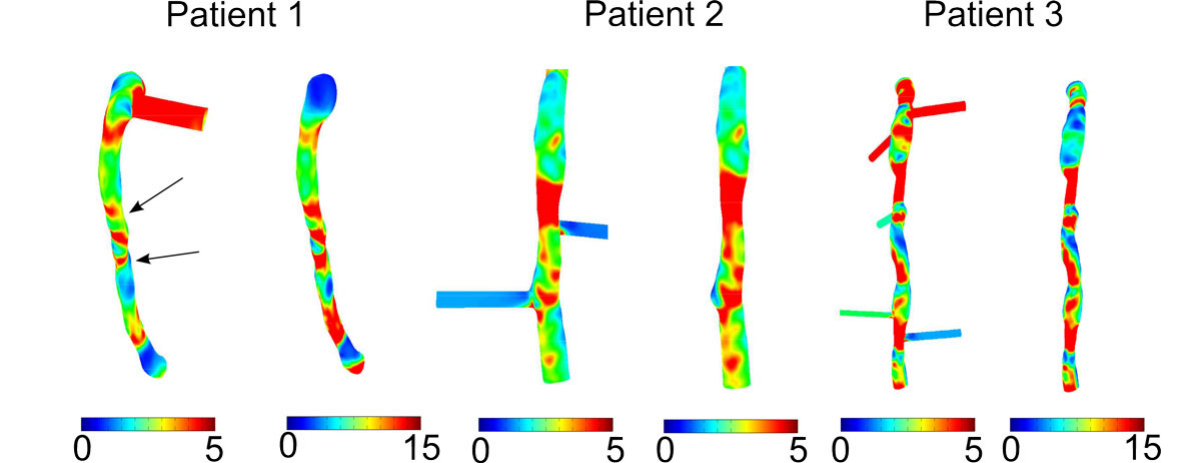
**Contour plots of time averaged WSS (Pa) for models with and without side-branches**. Arrows indicate where branches hidden by the view are located.

**Figure 3:**
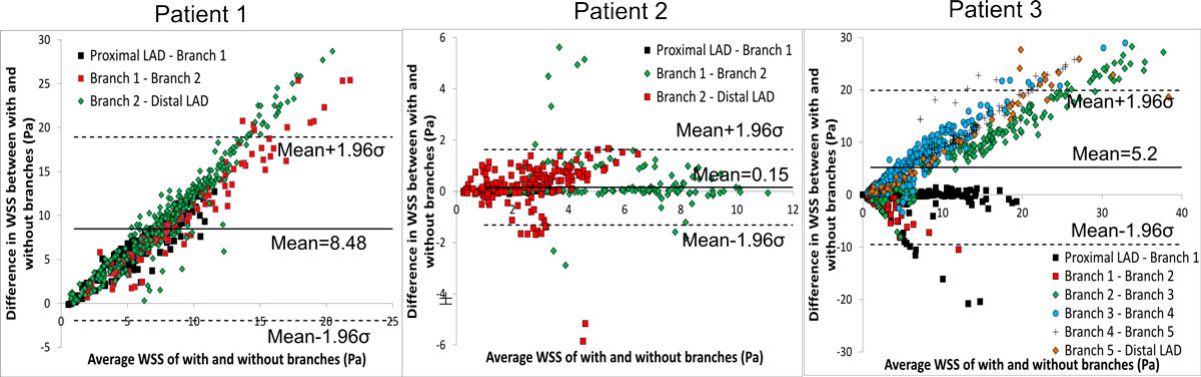
**Bland-Altman plots of differences in WSS between models with and without side-branches - patient 1 (left), patient 2(centre), patient 3 (right)**. The colors on the scatter plot show the region of the artery from which the calculated values are obtained. For example, Proximal LAD - Branch 1 indicates the difference in WSS between models in each sector between these 2 locations. The standard deviation is denoted by σ.

Time averaged WSS contour plots for each patient are shown in Figure 2. Qualitatively contours of WSS appear to be similar between models with and without branches in each patient; however, actual values can vary considerably between the models. Time averaged WSS values averaged over the entire LAD surface are presented in Table 2. Differences in the calculated WSS values between models with and without side-branches are shown in Bland-Altman plots in Figure 3. The largest differences were seen in patients 1 and 3 with mean differences of 8.5 ± 5.3 and 5.2 ± 7.5 Pa, respectively. Patient 2, which contains only 2 branches and a shorter LAD length, has a very small mean difference of 0.15 ± 0.75 Pa. In patients 1 and 3 there is a consistent bias of the WSS being greater in the models without side-branches, while this is not the case in patient 2. In patients 2 and 3 it can be observed that the smallest differences tend to occur in the proximal LAD prior to the first branch. This also appears to be the case in patient 1 despite hemodynamic differences in the proximal LAD section due to the presence of the LCX branch. In general, the further distal WSS is compared between both models the larger are the differences. Despite this, although patient 3 had the greatest number of branches, a greater mean difference was observed in patient 1.

**Table 2:** Time averaged WSS values averaged over model surface for each patient.

	WSS (Pa)	WSS without branches (Pa)	Difference (Pa)
1	3.2 ± 1.5	11.7 ± 6.4	8.5 ± 5.3
2	2.95 ± 2.3	3.1 ± 2.4	0.15 ± 0.75
3	5.5 ± 5.1	10.7 ± 11.3	5.2 ± 7.5

**Figure 4:**
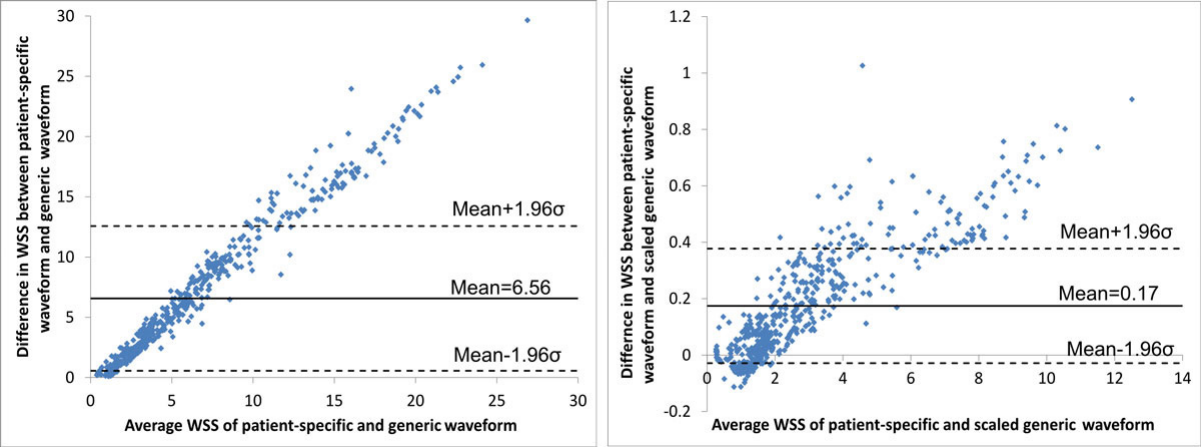
**Bland-Altman plots of differences in WSS between patient-specific waveforms and generic waveforms in patient 2 - patient-specific and generic (left), patient-specific and scaled generic (right)**. The standard deviation is denoted by σ.

Figure 4 shows the difference in calculated WSS between models with the patient-specific waveform and those with generic and scaled generic waveforms. The mean difference between the patient-specific and generic waveform was 6.56 ± 6 Pa. As both models contain side-branches in this case, this large difference can be solely attributed to the difference in input velocity. Unsurprisingly, due to the matched mean flow rates a much smaller change in WSS (0.17 ± 0.2 Pa) is observed between the patient-specific and scaled generic waveform.

**Figure 5:**
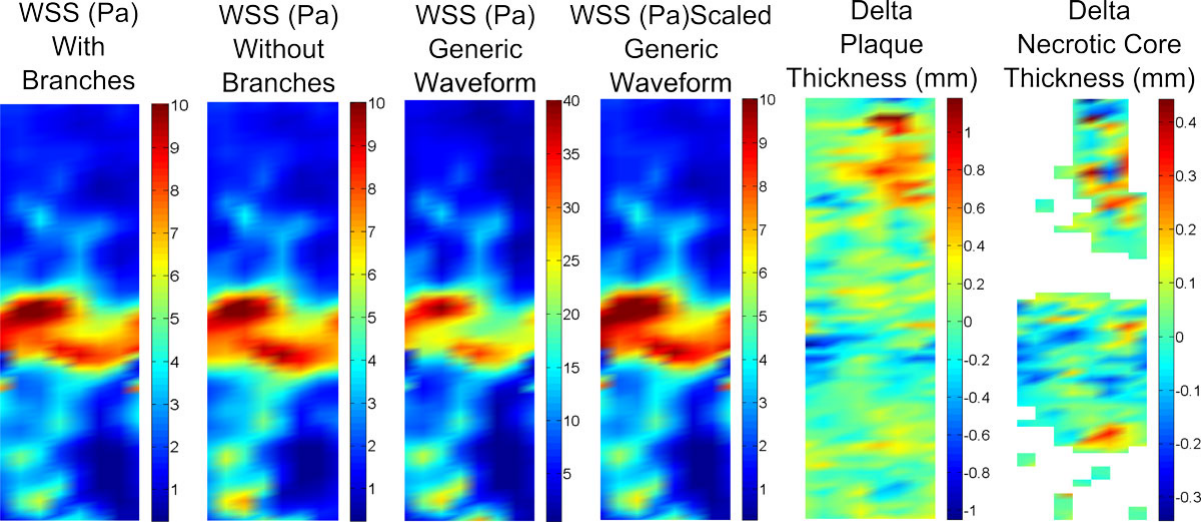
**Unwrapped plots for patient 2 from the proximal LAD (top) to the second side-branch (bottom)**. The first 4 plots show unwrapped WSS for different simulations (note the change in color scale for the plot with the generic waveform). The final 2 plots illustrate the change in plaque thickness and necrotic core thickness from baseline to follow-up.

Unwrapped plots of the geometry of patient 2 for WSS and plaque area changes can be seen in Figure 5. Qualitatively, WSS spatial patterns appear to be very similar among all simulations. However, there is a large difference in WSS magnitude between the model using the generic waveform and the other models, which can be attributed to the much greater input velocity in the generic waveform model. Also included in Figure 5 are the unwrapped plots of changes in plaque thickness and necrotic core thickness from baseline to follow up. An area of low WSS appears to co-localize with increases in plaque and necrotic core thickness in the proximal LAD, while a similar low WSS region in the distal vessel also shows an increase in necrotic core thickness and plaque thickness. However, there is also some plaque regression in this low WSS region.

### Changes in plaque components and wall shear stress

**Figure 6:**
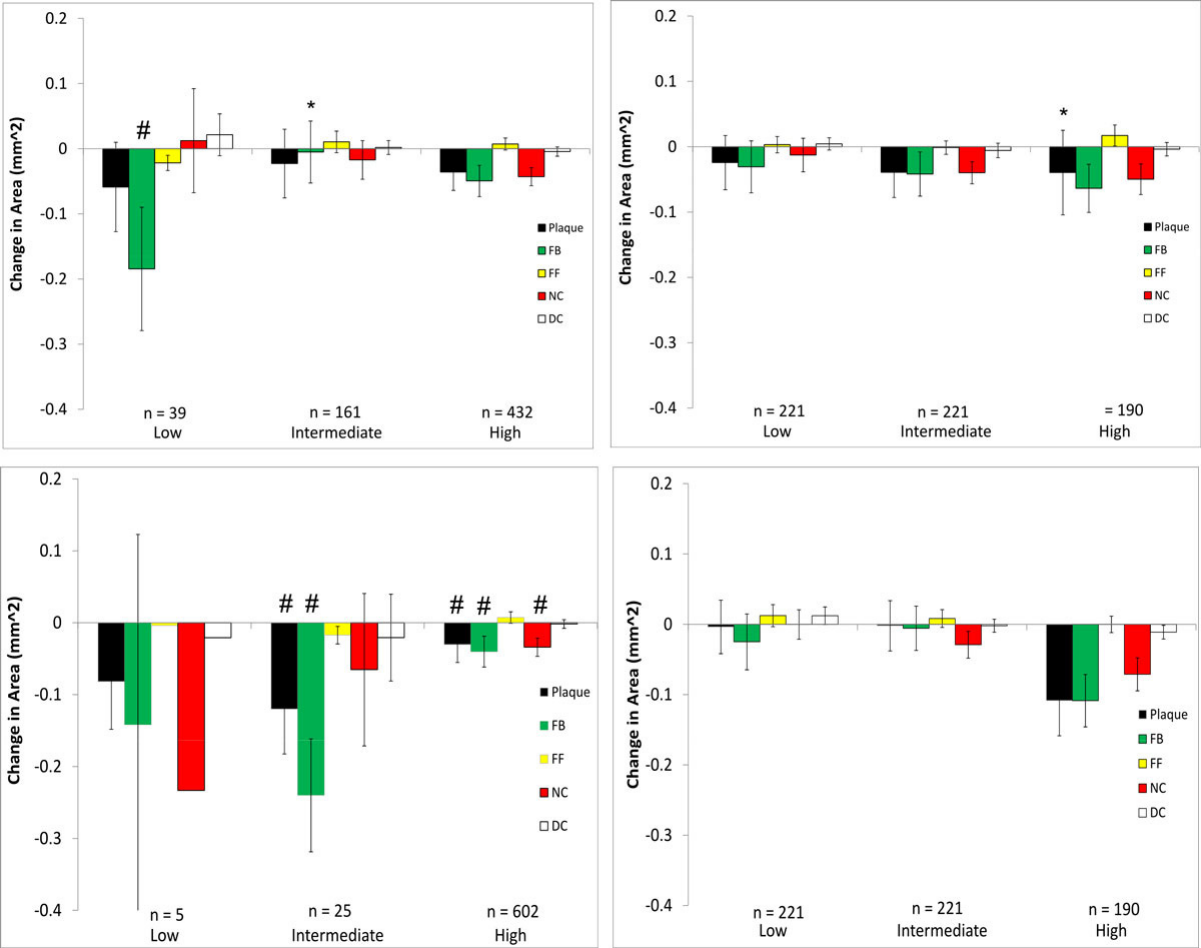
**Changes in VH-IVUS derived plaque component area in low-, intermediate-, and high WSS sectors in patient 1 for models with (top) and without (bottom) side-branches**. Data are further divided into WSS (left) and relative WSS (right), n refers to the number of plaque sectors in each WSS category (note, the number of sectors of any individual plaque component in a specific WSS category may differ from this as not each sector contains every plaque component). *P < 0.05 comparing the model with side-branches to the model without side-branches (top plot to bottom plot) waveform; # *P *< 0.05 comparing WSS to relative WSS differences (left plot to right plot)

**Figure 7:**
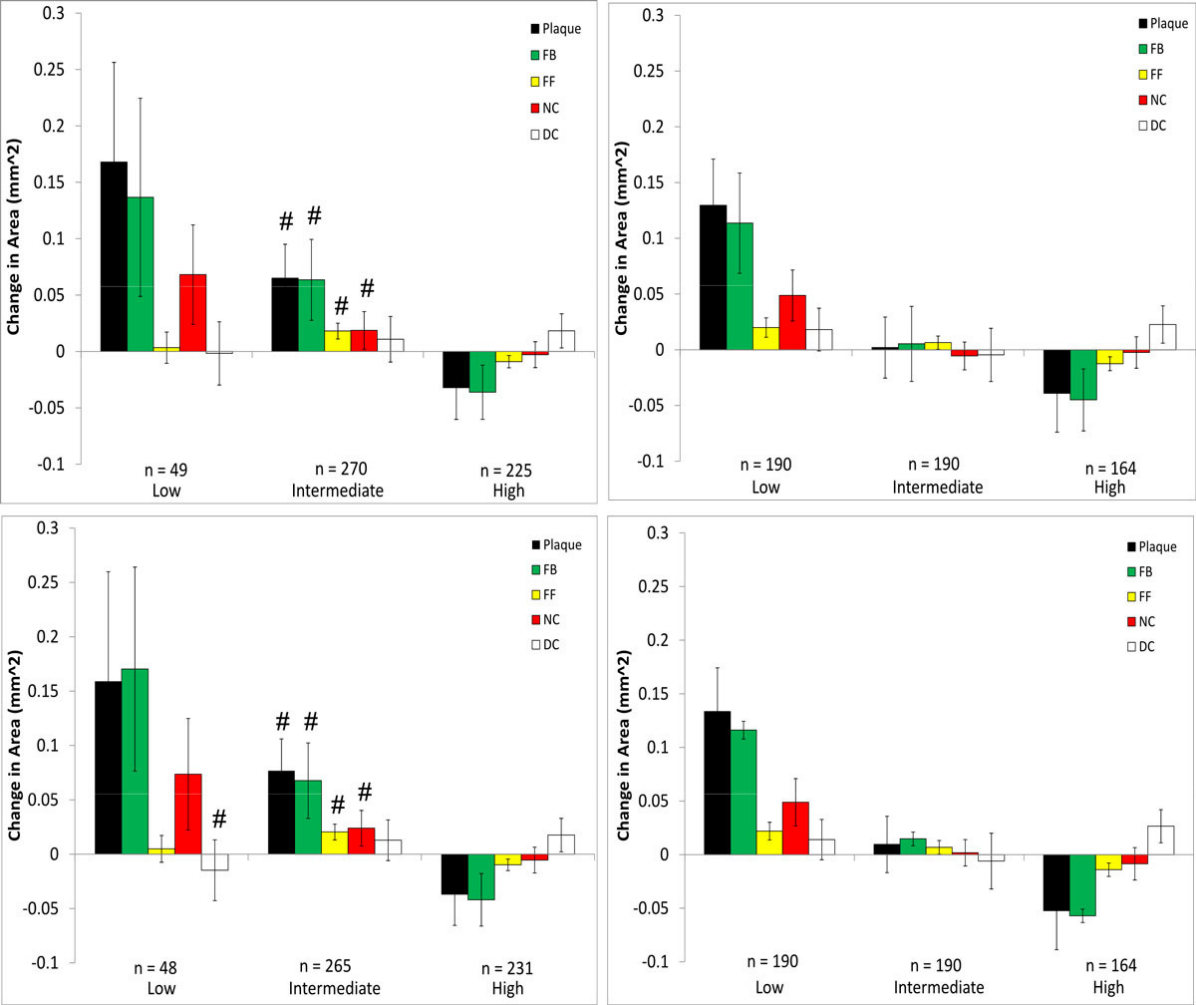
**Changes in VH-IVUS derived plaque component area in low-, intermediate-, and high WSS sectors in patient 2 for models with (top) and without (bottom) side-branches**. Data are further divided into WSS (left) and relative WSS (right), see Figure 5 for figure details.

**Figure 8:**
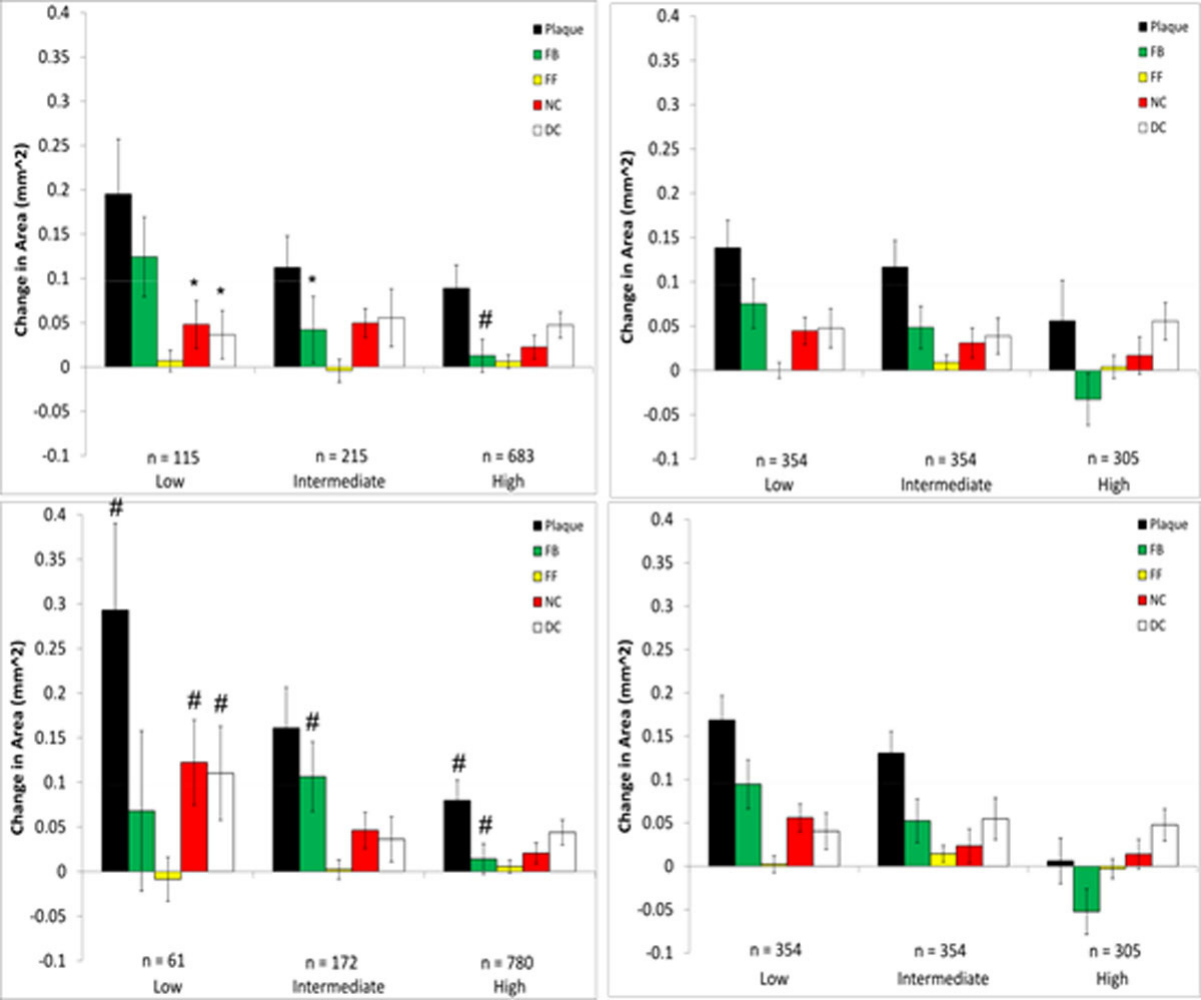
**Changes in VH-IVUS derived plaque component area in low-, intermediate-, and high WSS sectors in patient 3 for models with (top) and without (bottom) side-branches**. Data are further divided into WSS (left) and relative WSS (right), see figure 5 for figure detail.

**Figure 9:**
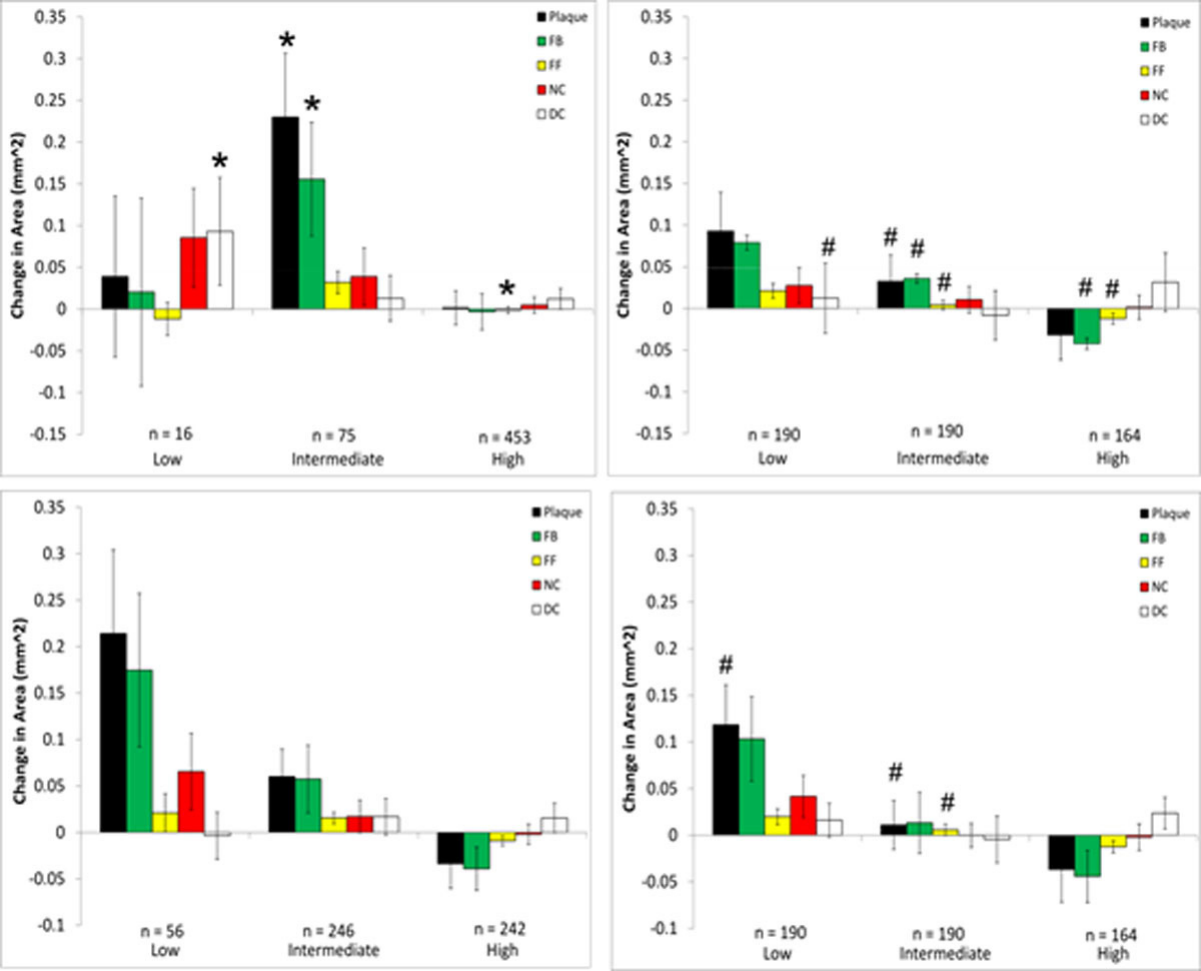
**Changes in VH-IVUS derived plaque component area in low-, intermediate-, and high WSS sectors in patient 2 for models using the generic waveform (top) and a scaled generic waveform (bottom)**. Data are further divided into WSS (left) and relative WSS (right). The corresponding patient-specific waveform data are in Figure 6. *P < 0.05 comparing patient-specific waveform to generic waveform # *P *< 0.05 comparing WSS to relative WSS differences (left to right).

Changes in plaque components from baseline to follow up were examined with respect to WSS at baseline for each model that was investigated, and the results are presented in Figures 6, 7, 8, 9 where the ordinate axes are total change in area divided by the number of sectors. Patient 1 was found to experience plaque regression during the 12 month period. As shown in Figures 2 and 3 the exclusion of side-branches resulted in computed WSS values that were, overall, larger than when side-branches were included. This was as expected because all sections of the model experienced the same flow rate due to the neglect of side-branch outflow. A consequence was that 602 sectors with plaque showed WSS > 2.5 Pa when branch outflow was eliminated, while only 432 sectors had WSS values > 2.5 Pa. Interestingly, the number of plaque sectors with low WSS (< 1 Pa) was reduced to only 5, so that the no side-branch assumption virtually eliminated sectors classified as low WSS; and only 25 sectors were classified as intermediate. There were very little noticeable changes in the high WSS category between the models. Differences in plaque change characteristics associated with WSS seen between the data for inclusion and exclusion of side branches were suppressed when presented using relative WSS categories, as seen in the right panels of Figure 6. The use of relative WSS creates a more even distribution of data amongst the sectors.

Somewhat in contrast to patient 1, patient 2 experienced plaque progression in low WSS sectors and plaque regression in high WSS sectors (Figure 7), regardless of how WSS was computed. No significant differences were observed whether side-branches were excluded or not in the relationship between plaque progression and all WSS categories. These findings agree very well with the Bland-Altman plot of patient 2 which also showed very little difference in WSS between these models. Characteristics of plaque change using relative WSS as a variable were similar to those using WSS, though there were differences in the intermediate WSS category.

All VH-IVUS components saw an increase in area in Patient 3. As with patient 2, there were similar characteristics for plaque changes in patient 3 between WSS models with and without side-branches and to a lesser extent between WSS and relative WSS (Figure 8). However, there were significant differences seen between models with and without side-branches in the low WSS category for necrotic core and dense calcium progression and also in the intermediate WSS category for fibrotic plaque. There were no significant differences in plaque progression association with relative WSS between models with and without side-branches. Several significant differences could be seen between plaque progression prediction using WSS and relative WSS, although this was almost completely confined to models without side-branches.

Large differences were seen in the association of plaque component changes with WSS when the generic waveform (See Figure 1) was used in patient 2 (Figure 9). This waveform results in a greater flow rate which creates higher WSS values, and as a result of this the number of values in the low WSS category is much smaller (n = 16) compared to the patient-specific (n = 49) or the scaled generic (n = 56) waveform. Use of the scaled generic waveform comes closer to replicating that of the patient-specific case, as there were no significant differences seen between both methods. Interestingly, when analyzed using relative WSS, both waveforms predict similar progression as compared to the patient-specific case (Figure 7). There were, however, several significant differences in plaque progression descriptions when analyzed using WSS versus relative WSS, particularly in the intermediate WSS category as was the case in the patient-specific waveform.

## Discussion

Many factors affect plaque localization and evolution, and mechanical factors are among these. There is considerable interest in employing CFD to compute WSS in coronary arteries and to relate this variable to changes in plaque composition over time, hopefully as one element of a clinical milieu that can be used to improve patient care. Understanding both the potential and limitations of WSS as a predictive variable is essential for addressing clinical needs. This study examined intra-patient variability in hemodynamics by assessing the focal association of WSS and atherosclerotic plaque changes over a twelve month period in three patients with coronary artery disease. Concentrating on flow-related boundary conditions for CFD, we explored the effects of altered patient coronary artery flow waveforms (inflow) and the presence or absence of side-branches (outflow). Given the potential differences these modelling strategies have on WSS values, we also investigated plaque changes using a relative WSS method. Since data acquisition typically occurs at one time point but there are temporal variations in flow rates and geometry over the course of a heartbeat and, more so, over longer time periods, caution may need to be exercised when relating plaque progression to actual, as opposed to relative, WSS values.

When comparing models with and without side-branches, greater differences in WSS values were seen in patients who had a greater number of side-branches. This is due to flow leaving the main vessel, resulting in a reduced distal flow rate and hence reduced WSS. Without these side-branches WSS remains artificially high in distal segments. For example, in patient 3 the presence of 5 side-branches resulted in a mean WSS difference of 5.2 Pa (Figure 3). Though patient 1 had only one more side-branch than patient 2, their respective differences in WSS for models with and without side-branches were 0.16 and 8.5 Pa. This can be attributed to the presence of the LCX branch in patient 1 which, due to its large size, has a large outflow. Similarly to the exclusion of side-branches, the use of a non patient-specific waveform with a high flow rate as the inflow condition resulted in a large mean difference (6.6 Pa). This difference was effectively eliminated when the same waveform was scaled to the same mean velocity as the patient-specific case (Figure 4), suggesting that the specific waveform shape is less important than the imposed mean flow. Qualitatively, the spatial patterns of relative WSS were similar regardless of the presence or absence of side branches or of the level of flow rate used as an input (Figure 5).

We then explored whether these hemodynamic differences would result in differences in interpreting plaque changes as related to WSS in individual patients. No significant differences were seen between inclusion/exclusion of side branches in patient 2 which had the least number of side branches and a shorter LAD length. On the other hand, more noticeable differences could be seen in patient 3 in low and intermediate WSS categories. This is due to the artificially higher distal flow rate caused by neglecting outflows in the five side-branches, thus increasing computed WSS and resulting in a greater number of sectors being categorized as high. For patient 1, despite the large hemodynamic differences seen, only fibrotic plaque change in the intermediate WSS category was statistically different between the side-branch/no side-branch cases. Large differences were seen in several of the components in the low WSS category, though as some of these (FF, NC, DC) contained only 1 sector they could not be tested for significance. Notably, neglecting branch outflow distorted the WSS field to the point that low WSS regions were essentially eliminated in patient 1.

The use of a generic inlet velocity waveform in patient 2 resulted in significant changes in plaque progression association with WSS compared to the use of the patient-specific waveform. This patient had an overall trend of increasing plaque and VH-IVUS component area in low WSS categories, changing to regression in high WSS sectors (Figure 7). While this trend was still evident in the scaled generic waveform model, the same could not be said for the basic generic waveform case. Scaling the generic waveform to match that of the mean velocity of the patient-specific waveform was seen to eliminate hemodynamic differences and as a result maintained the reference case relationships between changes in plaque component area and WSS.

Because of the sensitivity of computed values of WSS to inflow and outflow conditions, we also compared plaque changes in each scenario using a relative WSS metric under the hypothesis that areas of high WSS will remain relatively high and areas of low WSS will remain relatively low (this is more likely the case where the inlet waveform is altered as opposed to the side-branch exclusion cases, as there may be local changes in high and low WSS at these branch locations). When relative WSS is the hemodynamic variable being considered for plaque progression prediction, the generic waveform did not result in any significant differences compared to the patient-specific case (Figures 7 and 9). This is in contrast to using actual WSS where there were differences between the generic and patient-specific cases in observed plaque progression/WSS relationships. Using relative WSS also suppressed differences in observed plaque progression relationships when the models excluded side-branches (Figures 6, 7, 8, right panels). These findings suggest that relative WSS could be used as a surrogate for actual WSS in predicting plaque progression when patient-specific data are unavailable or unreliable.

A limitation of this study relates to how the outflow boundary conditions are treated. Ideally, patient pressure and flow rate should be measured in each branch, but this is not realistic in routine clinical practice. Previously, other investigators have specified outlet boundary conditions based on Murray's Law or empirical data from a study by Doriot et al. [15,16]. They reported that average WSS was higher by 8% when using Murray's Law, though the size of low WSS regions varied by up to 68% in bifurcation regions. Accurate knowledge of branch diameters is required in order to use these power laws, and these measurements were not available due to the limited penetration depth of IVUS. Variation of the flow rate under pressure free boundary conditions has previously been found to have no significant changes on predictive values of WSS [17]. Though we have not performed a sensitivity analysis of the boundary conditions in this study, we do not expect the uncertainty in outflow boundary conditions to have an important effect given that the WSS is ultimately parsed into low, intermediate and high categories.

## Conclusions

This study investigated the LAD coronary artery of patients with non-obstructive coronary artery disease and assessed effects on computed WSS arising from intra-patient variability in flow boundary conditions that may arise due to experimental measurement errors, flow changes to be expected under various physiological conditions, and assumptions made relating to the presence of side-branch outflow. The reference case for each individual employed patient-specific DUS velocity measurements from which LAD flow waveforms were computed, and the CFD model geometry included side-branches. Other cases examined were models without side-branches, and two alternative inlet flow waveforms.

Using VH-IVUS measurements of plaque components at baseline and 12 months follow-up in three patients, we describe changes in plaque area and in the areas of various plaque components, i.e., fibrotic, fibro-fatty, necrotic core, and dense calcium. We present quantitative observations of the relationship of changes in each plaque component with WSS as computed from the models, and we investigate the potential of a new variable - relative WSS - to improve the consistency of observations with respect to expected uncertainties in flow conditions. Based on these studies, we offer several conclusions:

1. Neglecting branch outflows can result in large differences in computed WSS (patients 1 and 3; Figures 2 and 3). Differences are exacerbated if the arterial segment being studied has multiple branches, as can be expected in longer segments.

2. Inlet flow waveform shape is not an important influence on computed WSS, although the overall mean flow level is a strong factor (patient 2; Figures 4 and 5).

3. The relationships between plaque component changes and computed WSS within a given patient can be significantly different depending upon the flow conditions. Specifically, neglecting side branches can lead to significantly different predictions of plaque progression than the reference case (patients 1 and 3; Figures 6 and 8, left panels) in individual cases.

4. High flow rates can also affect observed plaque progression relationships by comparison with the patient-specific flow rate case (patient 2; Figure 7, upper left panel, and Figure 9, upper left panel). This implies that use of a "snapshot" flow measurement at a baseline study may not fully capture this dynamic relationship experienced over periods of days or weeks.

5. Using relative WSS, rather than actual computed WSS, suppresses variability in describing plaque change relationships (patients 1,2,3; Figures 6, 7, 8, right panels).

These studies should be considered exploratory, since we investigated a small number of patients, and more extensive work is needed in order to develop definitive, optimum approaches that can give greater confidence in WSS/plaque prediction methods for individuals. Nonetheless, the present work serves to illustrate important issues that should be considered and demonstrates approaches that offer improvements in interpretation of data within the clinical setting.

## Competing interests

The authors declare that they have no competing interests.

## Ethics statement

All human data used in this study were obtained from consenting patients who presented to the Cardiac Catheterization Laboratory at Emory University Hospital and was approved by the hospital's Ethical Research Committee.

## Authors' contributions

DSM, LHT, HS and DPG designed the study. HS collected the patient data. DSM performed the reconstructions, simulations and drafted the manuscript. DSM, LHT, EA and DPG analyzed and interpreted the data. All authors read and reviewed the manuscript.

## Declarations

Publication of this article was paid with funding from the Georgia Research Alliance Eminent Scholar Program

This article has been published as part of *BioMedical Engineering OnLine *Volume 14 Supplement 1, 2015: Cardiovascular Disease and Vulnerable Plaque Biomechanics. The full contents of the supplement are available online at http://www.biomedical-engineering-online.com/supplements/14/S1
